# Ultrasound-Driven Healing: Unleashing the Potential of Chondrocyte-Derived Extracellular Vesicles for Chondrogenesis in Adipose-Derived Stem Cells

**DOI:** 10.3390/biomedicines11102836

**Published:** 2023-10-19

**Authors:** Yikai Wang, Zibo Liu, Chuqiao Pan, Yi Zheng, Yahong Chen, Xiang Lian, Yu Jiang, Chuhsin Chen, Ke Xue, Yuanyuan Zhang, Peng Xu, Kai Liu

**Affiliations:** 1 Shanghai Key Laboratory of Tissue Engineering, Department of Plastic and Reconstructive Surgery, Shanghai Ninth People’s Hospital, Shanghai Jiao Tong University School of Medicine, Shanghai 200011, China; wykpuck@sjtu.edu.cn (Y.W.); zibo_1999@sjtu.edu.cn (Z.L.); 823085@sh9hospital.org.cn (C.P.); justice08@sjtu.edu.cn (Y.Z.); 121042@sh9hospital.org.cn (Y.C.); lian_xiang1127@sjtu.edu.cn (X.L.); jiang-yu@sjtu.edu.cn (Y.J.); chenchuhsin@sjtu.edu.cn (C.C.); 118070@sh9hospital.org.cn (K.X.); 2 Wake Forest Institute for Regenerative Medicine, Wake Forest University Health Sciences, Winston-Salem, NC 27101, USA; yzhang@wakehealth.edu

**Keywords:** low-intensity ultrasound, chondrocyte-derived extracellular vesicles, adipose-derived stem cells, chondrogenesis, cartilage regeneration

## Abstract

Repairing cartilage defects represents a significant clinical challenge. While adipose-derived stem cell (ADSC)-based strategies hold promise for cartilage regeneration, their inherent chondrogenic potential is limited. Extracellular vesicles (EVs) derived from chondrocytes (CC-EVs) have shown potential in enhancing chondrogenesis, but their role in promoting chondrogenic differentiation of ADSCs remains poorly understood. Moreover, the clinical application of EVs faces limitations due to insufficient quantities for in vivo use, necessitating the development of effective methods for extracting significant amounts of CC-EVs. Our previous study demonstrated that low-intensity ultrasound (LIUS) stimulation enhances EV secretion from mesenchymal stem cells. Here, we identified a specific LIUS parameter for chondrocytes that increased EV secretion by 16-fold. CC-EVs were found to enhance cell activity, proliferation, migration, and 21-day chondrogenic differentiation of ADSCs in vitro, while EVs secreted by chondrocytes following LIUS stimulation (US-CC-EVs) exhibited superior efficacy. miRNA-seq revealed that US-CC-EVs were enriched in cartilage-regeneration-related miRNAs, contributing to chondrogenesis in various biological processes. In conclusion, we found that CC-EVs can enhance the chondrogenesis of ADSCs in vitro. In addition, our study introduces ultrasound-driven healing as an innovative method to enhance the quantity and quality of CC-EVs, meeting clinical demand and addressing the limited chondrogenic potential of ADSCs. The ultrasound-driven healing unleashes the potential of CC-EVs for chondrogenesis possibly through the enrichment of cartilage-regeneration-associated miRNAs in EVs, suggesting their potential role in cartilage reconstruction. These findings hold promise for advancing cartilage regeneration strategies and may pave the way for novel therapeutic interventions in regenerative medicine.

## 1. Introduction

Repairing cartilage defects remains a clinical challenge due to their limited intrinsic healing capacity [1]. The limited regenerative potential of cartilage is often attributed to the low cell density and the limited proliferative capacity of mature chondrocytes [2]. Current treatment modalities for cartilage defects, including microfractures and autologous chondrocyte implantation, often result in fibrocartilage formation or cartilage hypertrophy, making it difficult to achieve satisfactory repair outcomes [3,4].

In recent years, mesenchymal stem cell-based strategies and cartilage tissue engineering have emerged as promising modalities for cartilage regeneration [5,6]. Bone marrow-derived stem cells (BMSCs) are the major cell types involved in cartilage defect repair [7]. Although BMSCs exhibit strong chondrogenic abilities [8], their acquisition process is invasive [9]; during in vitro chondrogenesis, they are prone to hypertrophy, forming fibrotic cartilage [10]. Adipose-derived stem cells (ADSCs) can be isolated in relatively large quantities through less invasive and minimally painful procedures compared to BMSCs [11]. In addition, the proliferative and differentiation capacities of ADSCs are unlikely to be affected by donor age [12]. Previous studies have demonstrated that ADSCs can differentiate into chondrocytes; however, they exhibit lower chondrogenic potential than BMSCs [13,14]. This limited chondrogenic capacity hinders the applicability of ADSCs in cartilage regeneration.

Recent reports have shown that a paracrine mechanism plays a significant role in intercellular communication, mediating various biological effects, including angiogenesis, wound healing promotion, and inflammation regulation [15,16,17]. Extracellular vesicles (EVs) are the primary paracrine mediators and contain biological information on the cells from which they originate [15]. Chondrocyte-derived extracellular vesicles (CC-EVs) were reported to promote chondrogenesis, including promoting the cartilage differentiation of progenitor cells [18] and regulating the catenin pathway to promote the chondrogenic differentiation of BMSCs [19]. Therefore, we hypothesize that their application may improve the quality of cartilage repair using ADSCs.

Currently, CC-EVs are primarily obtained from the supernatants of chondrocyte cultures. However, chondrocytes are highly differentiated cells with limited regenerative capacity, and they face the challenge of phenotypic dedifferentiation after multiple passages in culture [20]. The availability of chondrocyte sources is limited, resulting in limited access to chondrocyte-derived EVs. To utilize chondrocyte-derived EVs for treating cartilage defects, developing a method that extracts significant quantities of EVs from chondrocytes is crucial.

Recently, low-intensity ultrasound (LIUS) has emerged as an innovative tool in the field of regenerative medicine [21]. Since a clinical trial in 1994 demonstrated that LIUS can accelerate the fracture repair process [22,23], its efficacy has been extended to the regeneration of various tissues. LIUS promotes cartilage regeneration in various ways, including alleviating chondrocyte damage, promoting chondrocyte proliferation and differentiation, and promoting MSC-transplantation-based articular cartilage regeneration [24,25,26,27]. While chondrocytes are the predominant cell type within cartilage, there has been limited research on whether LIUS could affect the secretion of EVs by chondrocytes. Our previous study revealed that LIUS promotes ADSCs to secret more EVs [28]. Inspired by these findings, we explored whether LIUS could be applied to chondrocytes to stimulate EV secretion and enhance their ability to promote chondrogenesis of ADSCs.

Therefore, we initially identified the appropriate low-intensity ultrasound parameters to enhance the secretion of EVs by chondrocytes. Subsequently, we investigated the biological effects of CC-EVs and EVs derived from chondrocytes stimulated with LIUS (US-CC-EVs) on ADSCs in vitro, particularly their potential to promote chondrogenic differentiation. Additionally, through miRNA sequencing of CC-EVs and US-CC-EVs, we investigated the mechanisms underlying the enhanced chondrogenesis effects of CC-EVs following ultrasound. In summary, we found that CC-EVs can enhance the chondrogenesis of ADSCs in vitro. In addition, this study introduces ultrasound-driven healing as an innovative method to enhance the quantity and quality of CC-EVs.

## 2. Results

### 2.1. Characterization of ADSCs

The morphology of ADSCs exhibited a spindle-like morphology (Figure S1A). Moreover, to evaluate the multipotency of ADSCs, they were cultured in different induction media to induce chondrogenic, adipogenic, and osteogenic differentiation, respectively. The images obtained after Alizarin Red, Oil Red O, and Alcian Blue staining (Figure S1B) demonstrated that ADSCs differentiated into multiple lineages. Specific markers of ADSCs were detected by flow cytometry. ADSCs were positive for the typical stem cell surface markers CD29, CD90, and CD105 but negative for CD31, CD34, and CD45 (Figure S1C).

### 2.2. Optimization of Low-Intensity Ultrasound Parameters

**Figure 1:**
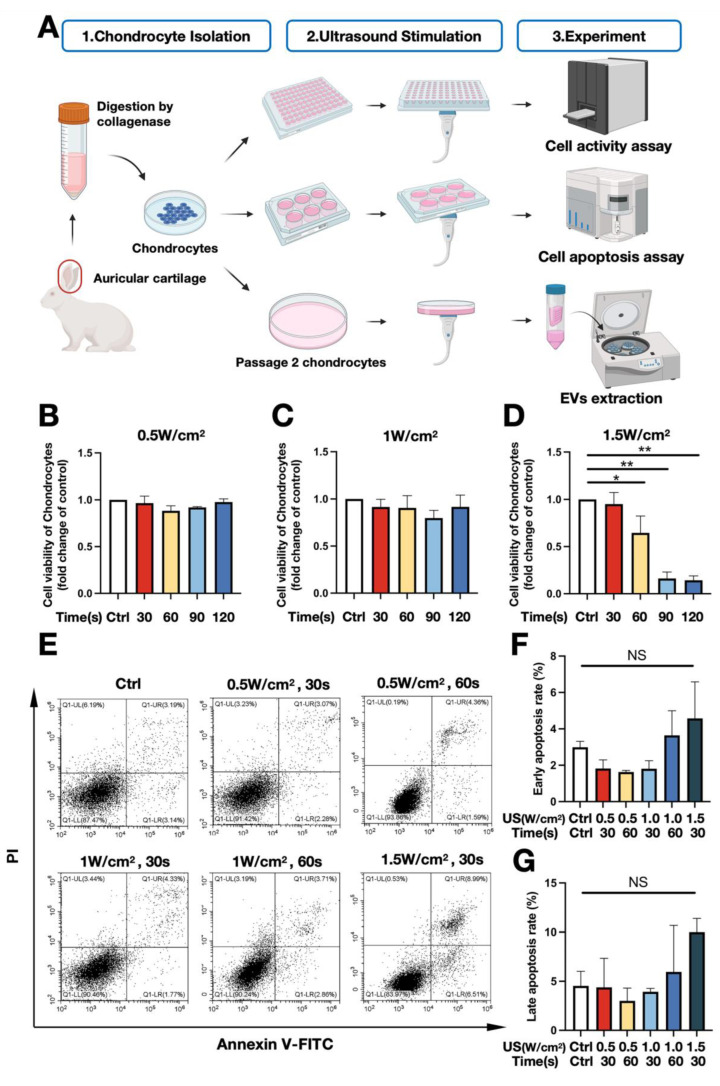
Optimization of low-intensity ultrasound parameters. (**A**) Illustration of the experiment. (**B**–**D**) Chondrocyte viability after different LIUS stimulation parameters on day 3 using Cell Counting Kit-8 (n = 3). (**E**) Chondrocyte apoptosis following LIUS stimulation at 48 h detected by flow cytometry. (**F**,**G**) Analysis of chondrocyte apoptosis; (**F**) early apoptosis rate (n = 3); (**G**) late apoptosis rate (n = 3). * *p* < 0.05; ** *p* < 0.01; NS: no significant difference.

Initially, we selected low-intensity ultrasound (LIUS) intensities of 0.5, 1.0, and 1.5 W/cm^2^ and stimulation times of 30, 60, 90, and 120 s based on our previous study [28]. To further explore the suitability of LIUS for chondrocytes, we assessed cell viability using different ultrasound parameters. Three days after ultrasound stimulation, the CCK-8 assay results showed that chondrocyte viability did not significantly change with different stimulation times at 0.5 and 1.0 W/cm^2^ (Figure 1B,C). However, an apparent decrease in cell viability was observed when the chondrocytes were treated for 60 (*p* < 0.05), 90 (*p* < 0.01), and 120 s (*p* < 0.01) at an intensity of 1.5 W/cm^2^ (Figure 1D); to reduce chondrocyte damage and minimize treatment time, we selected 0.5 and 1.0 W/cm^2^ for 30 and 60 s respectively, and 1.5 W/cm^2^ for 30 s for further exploration.

Apoptosis assays were conducted to investigate whether low-intensity ultrasound induces chondrocyte apoptosis. “FITC positive, PI negative” indicates early-stage apoptosis, while “FITC positive, PI positive” represents late-stage apoptosis (Figure 1E). No statistically significant variations were observed in the rates of early and late apoptosis among the different groups (Figure 1E–G); although 1.5 W/cm^2^ for 30 s slightly increased the late apoptosis rate, the difference was not statistically significant (*p* > 0.05) (Figure 1G). By collective consideration, we selected intensities of 0.5, 1.0, and 1.5 W/cm^2^ with a stimulation time of 30 s (which caused minimal cell damage and had the shortest processing time) for further experiments.

### 2.3. Appropriate Low-Intensity Ultrasound Significantly Promotes the Release of Extracellular Vesicles by Chondrocytes

**Figure 2:**
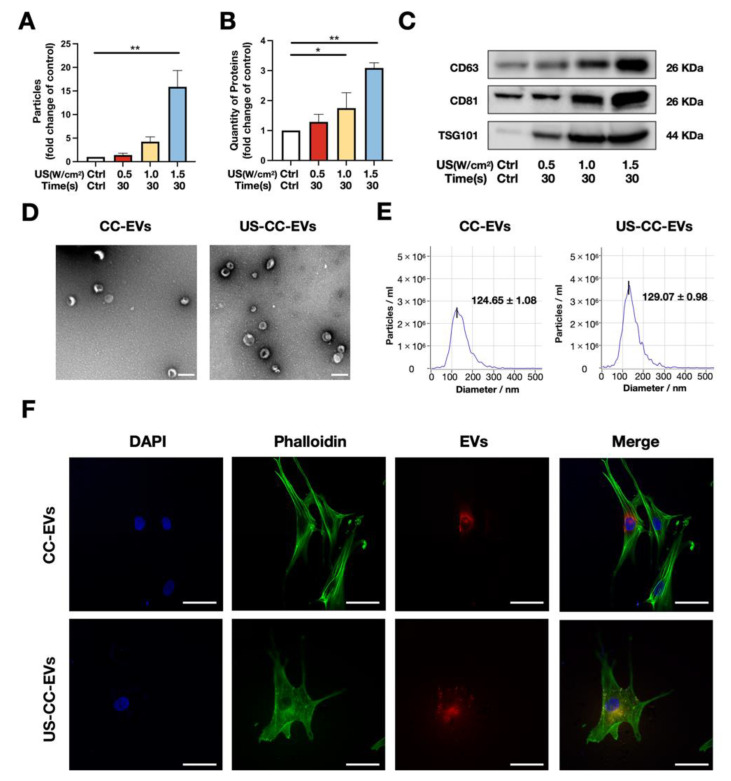
Low-intensity ultrasound stimulation promoted chondrocyte extracellular release. (**A**) Particle numbers of EVs following different ultrasound stimulation parameters detected by NTA (n = 3). (**B**) Protein quantity of EVs following different ultrasound stimulation parameters detected by BCA protein assay (n = 3). (**C**) Western blotting for EV-related markers CD63, CD81, and TSG101. (**D**) Morphological characterization of the EVs derived from chondrocytes (CC-EVs) without ultrasound stimulation and EVs derived from chondrocytes with 1.5 W/cm^2^ and 30 s ultrasound stimulation (US-CC-EVs) via transmission electron microscopy; scale bar = 200 nm; (**E**) Particle diameter distribution of CC-EVs (median 124.65 ± 1.08 nm) and US-CC-EVs (median 129.07 ± 0.98 nm) detected by NTA; (**F**) Uptake of CC-EVs and US-CC-EVs in ADSCs detected by confocal microscopy; scale bar = 50 μm. NTA: nanoparticle tracking analysis; * *p* < 0.05; ** *p* < 0.01.

EVs derived from chondrocytes stimulated with ultrasound were isolated from conditioned media using an ultrafiltration method and analyzed using nanoparticle tracking analysis (NTA), bicinchoninic acid (BCA) protein assay, and western blotting. The NTA results showed that the 1.5 W/cm^2^ for 30 s parameter stimulated chondrocytes to secrete >16-fold more EVs (*p* < 0.01) than chondrocytes without ultrasound treatment (control group). In contrast, no significant differences were observed between the control group and the chondrocytes stimulated with the 0.5 W/cm^2^ for 30 s and 1.0 W/cm^2^ for 30 s ultrasound parameters (Figure 2A). The BCA protein assay results showed that the 1.0 W/cm^2^ for 30 s parameter increased the protein quantity of EVs by 1.74-fold (*p* < 0.05), and the 1.5W/cm^2^ for 30 s parameter raised the protein quantity of EVs by 3.08-fold (*p* < 0.01) compared to the control group. Conversely, no significant differences were observed between the control group and the chondrocytes stimulated with 0.5 W/cm^2^ for 30 s (Figure 2B). In addition, western blot analysis was performed on EVs collected from chondrocytes treated with different ultrasound parameters to assess the expression of EV marker proteins, including CD63, CD81, and TSG101. The levels of these EV marker proteins were higher in EV samples obtained from ultrasound-treated chondrocytes than in the control samples, indicating enhanced secretion of EVs in response to ultrasound stimulation (Figure 2C). As determined by the EV number and total EV protein quantity, more EVs were released from the same number of chondrocytes treated with 1.5 W/cm^2^ for 30 s.

As shown in TEM results (Figure 2D), the EVs derived from chondrocytes (CC-EVs) and CC-EVs stimulated with the 1.5 W/cm^2^ for 30 s ultrasound (US-CC-EVs) exhibited typical cup-shaped morphology with a diameter of 124.65 ± 1.08 nm (CC-EVs) and 129.07 ± 0.98 nm (US-CC-EVs) (Figure 2E). After incubation with ADSCs for 12 h, we observed that CC-EVs and US-CC-EVs were internalized by ADSCs, and there were no significant differences in the internalization of the two types of EVs in ADSCs (Figure 2F). These results confirmed that 1.5 W/cm^2^ for 30 s ultrasound stimulation significantly promoted the release of EVs by chondrocytes without causing apparent cellular damage or apoptosis.

### 2.4. CC-EVs and US-CC-EVs Promote Cell Activity, Proliferation, and Migration of ADSCs

**Figure 3:**
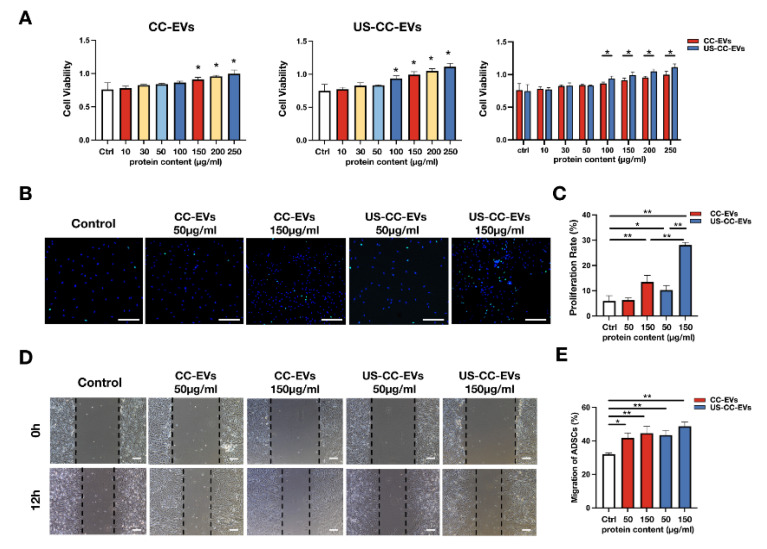
CC-EVs and US-CC-EVs promoted cell activity, proliferation, and migration of ADSCs. (**A**) Cell viability of ADSCs incubated with different concentrations of CC-EVs and US-CC-EVs using Cell Counting Kit-8 (n = 3). (**B**) Cell proliferation as determined by the EdU assay; EdU-positive ADSCs (green) and cell nucleus (blue) were observed using fluorescence microscopy. Scale bar = 500 μm. (**C**) ADSCs proliferation rate (n = 4). (**D**) Wound healing assay of ADSCs observed using a light microscope; scale bar = 200 μm. (**E**) ADSCs migration rate (n = 3). * *p* < 0.05; ** *p* < 0.01.

To assess the effects of CC-EVs and US-CC-EVs on ADSCs activity, ADSCs were incubated with different CC-EVs and US-CC-EVs. After 72 h, CCK-8 results showed that US-CC-EVs significantly (*p* < 0.05) promote ADSCs cell viability at concentrations of 100 to 250 μg/mL. However, CC-EVs only enhanced (*p* < 0.05) ADSCs cell viability at concentrations ranging from 150 to 250 μg/mL; at a concentration of 100 μg/mL, CC-EVs did not significantly promote cell viability (*p* > 0.05) (Figure 3A). In addition, the 100, 150, 200, and 250 μg/mL US-CC-EVs groups showed higher cell viability than CC-EVs groups (*p* < 0.05) (Figure 3A). Collectively, CC-EVs and US-CC-EVs concentrations of 150 μg/mL (which significantly promoted ADSCs cell viability) and a low concentration of 50 μg/mL were selected for subsequent experiments.

ADSCs proliferation was evaluated using the EdU assay. At a concentration of 150 μg/mL, CC-EVs and US-CC-EVs stimulated ADSCs proliferation when compared to the control group (*p* < 0.01); furthermore, US-CC-EVs had a stronger effect on ADSCs proliferation than CC-EVs (*p* < 0.01) (Figure 3B,C). Notably, at a concentration of 50 μg/mL, only US-CC-EVs promoted ADSCs proliferation (*p* < 0.05) (Figure 3B,C).

Scratch wound healing assays were performed to investigate cell migration; CC-EVs and US-CC-EVs of 50 μg/mL and 150 μg/mL significantly accelerated ADSCs migration (*p* < 0.05), without significant difference between them at the same concentration (Figure 3D,E). These results demonstrated that CC-EVs and US-CC-EVs enhance cell viability, proliferation, and migration of ADSCs, with US-CC-EVs exerting a stronger effect on cell viability and proliferation.

### 2.5. CC-EVs and US-CC-EVs Promote the Chondrogenic Differentiation of ADSCs

**Figure 4:**
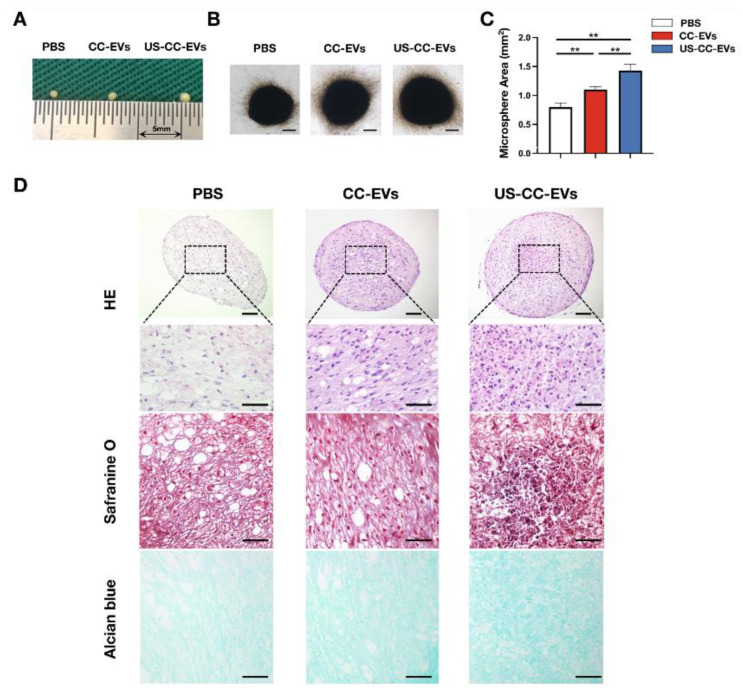
CC-EVs and US-CC-EVs promoted the chondrogenic differentiation of ADSCs. (**A**) Macroscopic photo. (**B**) Microscopic photo; scale bar = 250 μm. (**C**) Area measured by a light microscope using ImageJ (n = 3). (**D**) Histological assessment by Hematoxylin and Eosin (H&E), Safranine O, Alcian blue staining at 21 days of chondrogenic differentiation in micromass culture of ADSCs; scale bar = 200 μm (first row); scale bar = 100 μm (last three rows). ** *p* < 0.01.

To explore the potential role of CC-EVs and US-CC-EVs in chondrogenic differentiation, ADSCs were subjected to chondrogenic differentiation in a chondrogenic medium using micromass culture (an in vitro cultivation method with high-density seeding) and treated with phosphate-buffered saline (PBS), CC-EVs, or US-CC-EVs. After 21 days of chondrogenesis, the cell microspheres treated with CC-EVs or US-CC-EVs (150 μg/mL) were significantly larger than those in the PBS group. Furthermore, the cell microspheres treated with US-CC-EVs were larger than those treated with CC-EVs (*p* < 0.05) (Figure 4A–C). Hematoxylin and eosin (H&E) staining results showed that the cell microspheres treated with US-CC-EVs exhibited the most compact tissue structure with the least number of internal cavities (Figure 4D). Safranine O and Alcian blue staining were used to detect glycosaminoglycan (GAG) deposition. The CC-EVs and US-CC-EV groups exhibited more intense staining than the PBS group, indicating that treatment with CC-EVs or US-CC-EVs enhanced the deposition of GAG. The US-CC-EV-treated group exhibited the highest level of GAG deposition (Figure 4D).

**Figure 5:**
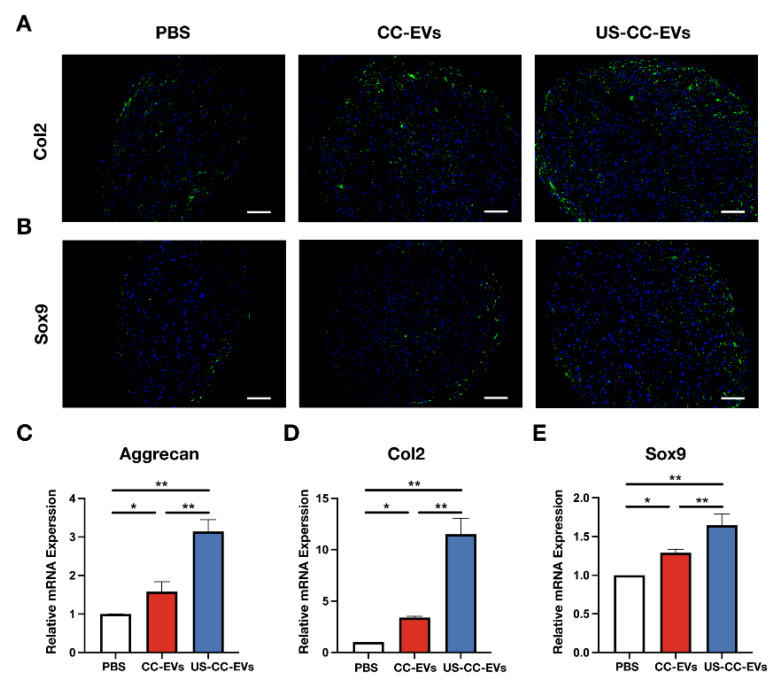
CC-EVs and US-CC-EVs promoted the chondrogenic differentiation of ADSCs. (**A**,**B**) Immunofluorescence images of Col2 and Sox9 protein accumulation; scale bar = 200 μm. (**C**–**E**) mRNA expression levels of Aggrecan, Col2, and Sox9 were measured by qPCR at 21 days of chondrogenic differentiation in micromass culture of ADSCs. * *p* < 0.05; ** *p* < 0.01.

Consistently, the results of immunofluorescence staining of Col2 and Sox9 demonstrated that the expression of Col2 and Sox9 was higher than that in the PBS group at 21 days in the CC-EVs and US-CC-EVs groups (Figure 5A,B). In addition, the US-CC-EVs group showed the highest levels of Col2 and Sox9 expression (Figure 5A,B). Stimulation with CC-EVs or US-CC-EVs upregulated the mRNA expression levels of genes related to chondrogenesis, such as Aggrecan, Col2, and Sox9, which is consistent with the immunofluorescence staining results (*p* < 0.05) (Figure 5C–E). Furthermore, the US-CC-EV group exhibited higher mRNA expression levels than the CC-EVs group (*p* < 0.05) (Figure 5C–E).

Collectively, these data suggest that CC-EVs and US-CC-EVs contribute to the chondrogenic differentiation of ADSCs and that US-CC-EVs are more beneficial than CC-EVs.

### 2.6. Expression Profiling of CC-EV and US-CC-EV miRNAs

**Figure 6:**
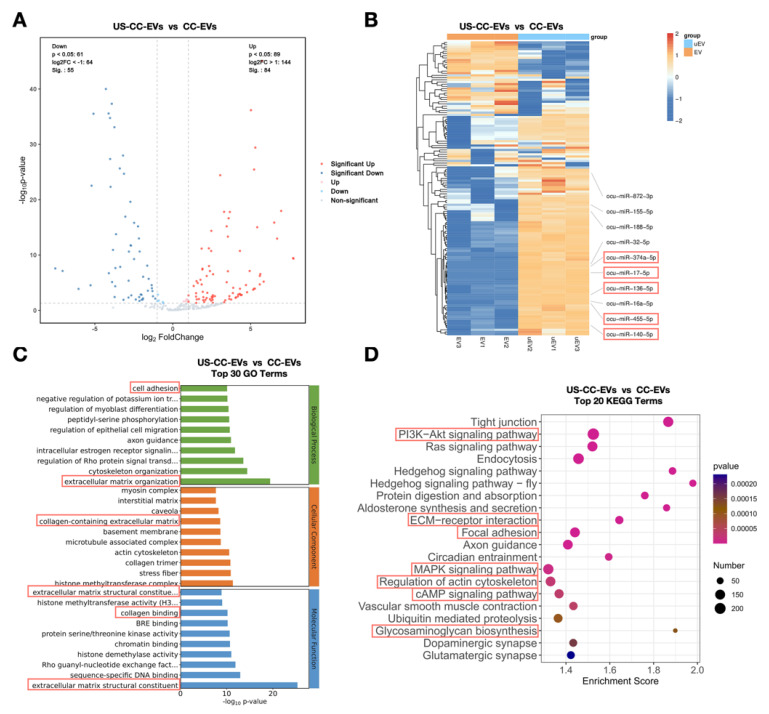
US-CC-EVs exhibited an enrichment of miRNAs associated with chondrogenic differentiation after low-intensity ultrasound stimulation. (**A**) Volcano plot of differentially expressed miRNAs between US-CC-EVs and CC-EVs (n = 3). Red dots: significantly upregulated miRNAs; blue dots: significantly downregulated miRNAs. (**B**) Hierarchical clustering analysis of differentially expressed miRNAs (|log_2_foldchange| > 1 and q-value < 0.05; unreported miRNAs were omitted) between US-CC-EVs and CC-EVs. Rows represent miRNAs; columns represent individual replicates. The top ten miRNAs with the high fold change are annotated, and miRNAs associated with chondrogenic differentiation are highlighted. (**C**) Gene Ontology (GO) analysis of differentially expressed genes predicted based on the differentially expressed miRNAs. Terms associated with chondrogenic differentiation are highlighted. (**D**) Kyoto Encyclopedia of Genes and Genomes (KEGG) pathway analysis of differentially expressed genes predicted based on the differentially expressed miRNAs. Terms associated with chondrogenic differentiation are highlighted.

MiRNA-seq was conducted on US-CC-EVs and CC-EVs to elucidate the underlying mechanisms by which ultrasound enhances the biological effects of EVs. Sequencing results revealed 84 upregulated and 55 downregulated miRNAs in chondrocyte-derived EVs following ultrasound stimulation (Figure 6A) (|log_2_foldchange| > 1, q-value < 0.05, unreported miRNAs were omitted). Hierarchical cluster analysis was performed on differentially expressed miRNAs (the upregulated miRNAs); the top ten miRNAs with high fold changes were annotated and searched in PubMed. Half of the top ten upregulated miRNAs, namely, miR-374a-5p, miR-17-5p, miR-136-5p, miR-455-5p, and miR-140-5p (highlighted), have been reported to enhance cartilage regeneration (Figure 6B).

To better understand the role of the differentially expressed miRNAs between US-CC-EVs and CC-EVs, the predicted target genes of the differentially expressed miRNAs were analyzed, and Gene Ontology (GO) analysis was conducted to understand their functional roles. The top 10 GO terms in the categories of “biological process,” “cellular component,” and “molecular function” are presented. GO analysis revealed that differentially expressed miRNAs were involved in several terms related to cartilage regeneration, such as “extracellular matrix organization, cell adhesion, collagen-containing extracellular matrix, extracellular matrix structural constituents, collagen binding,” and “extracellular matrix structural constituents “ (Figure 6C). KEGG analysis revealed that the differentially expressed miRNAs were associated with specific pathways that have been reported to be closely related to cartilage: “focal adhesion,” “regulation of actin cytoskeleton,” “ECM-receptor interaction,” “PI3K-AKT signaling pathway,” “cAMP signaling pathway,” “MAPK signaling pathway,” and “glycosaminoglycan biosynthesis-heparan sulfate/heparin” (Figure 6D). These data indicated that US-CC-EVs were enriched in cartilage-regeneration-related miRNAs contributing to chondrogenesis in various biological processes.

## 3. Discussion

Repairing cartilage defects represents a significant clinical challenge. While adipose-derived stem cell (ADSC)-based strategies hold promise for cartilage regeneration, their inherent chondrogenic potential is limited. Here, we found that CC-EVs can enhance the chondrogenesis of ADSCs in vitro. In addition, we introduce ultrasound-driven healing as an innovative method to enhance the quantity and quality of CC-EVs.

Previous research has indicated that ultrasound, when applied with different parameters, elicits varying biological effects in different tissues or cells [29]. High-intensity ultrasound primarily exerts its effects through a thermal mechanism [30], whereas low-intensity ultrasound leads to non-thermal effects, which lead to therapeutic applications [24]. Although our previous research demonstrated that low-intensity ultrasound can stimulate ADSCs to release EVs, whether low-intensity ultrasound can increase the secretion of extracellular vesicles by chondrocytes had not been studied. Based on our previous experiments, we initially applied ultrasound to chondrocytes using intensities ranging from 0.5 to 1.5 W/cm^2^ and exposure times ranging from 30 to 120 s. With the increase in ultrasound intensity and extended exposure time, LIUS, a form of mechanical energy, enhances its thermal and non-thermal effects on cells [31]. To prevent irreversible damage to chondrocytes, we assessed the viability and apoptotic rates of chondrocytes. Based on the above experiments, we selected 0.5 W/cm^2^, 1.0 W/cm^2,^ and 1.5 W/cm^2^ and the shortest ultrasound duration of 30 s to further assess the secretion of extracellular vesicles following ultrasound stimulation. It is worth noting that although ultrasound stimulation with 1.5 W/cm^2^ for 30 s slightly increased the late-stage apoptotic rate, the difference was not statistically significant. Considering apoptotic cell-derived EVs are vital in many biological regulations, such as facilitating the osteogenic differentiation of BMSCs [32] and maintaining bone homeostasis [33], we did not exclude the ultrasound parameters of 1.5 W/cm^2^ for 30 s.

Through NTA, BCA, and western blot analyses, we confirmed that ultrasound stimulation at 1.5 W/cm^2^ for 30 s among the three ultrasound parameters showed the most significant promotion of EV secretion in chondrocytes; these EVs, referred to as US-CC-EVs, exhibited a typical EV shape, diameter, and specific marker expression. In addition, there were no significant differences between the ADSCs internalization of CC-EVs and US-CC-EVs. The particle number of EVs increased 16-fold, and the protein content increased 3-fold using our ultrasound-driven healing. In our previous study, the application of the same ultrasound parameters to ADSCs resulted in a 45-fold increase in particle quantity and a 3.1-fold increase in protein quantity compared with the control group [28]. However, when ultrasound was applied to chondrocytes to promote EV secretion, the effect was relatively weak; this could be because chondrocytes are relatively inert compared to mesenchymal stem cells [34]. Previously reported EV production methods that rely on global cellular stress responses, such as a stiff matrix (3-fold) [35], hypoxia (1.44-fold) [36], and heat treatment (2.3-fold) [37], resulted in moderate EV release. Notably, our ultrasound-driven healing as an innovative method to enhance the production of chondrocyte-derived vesicles, demonstrated a significantly higher increase in EV production than previously reported methods. However, the mechanism by which LIUS promotes the secretion of extracellular vesicles by chondrocytes remains to be elucidated.

CC-EVs have shown potential in enhancing chondrogenesis, but their role in promoting chondrogenic differentiation of ADSCs remains poorly understood. So, we evaluated the biological and chondrogenesis effects of CC-EVs in ADSCs. In addition, LIUS has been shown to alleviate chondrocyte damage in osteoarthritis [38] and enhance the regenerative potential of BMSC-derived extracellular vesicles in promoting cartilage regeneration [39]. Next, we aim to demonstrate whether our ultrasound-driven healing as an innovative method to enhance the production of chondrocyte-derived vesicles can enhance the ability of EVs to promote chondrogenesis in ADSCs. Results from the CCK-8, EdU incorporation, and scratch wound healing assays indicated that CC-EVs and US-CC-EVs enhanced the viability, proliferation, and migration of ADSC, with US-CC-EVs exerting a stronger effect on cell viability and proliferation. Subsequently, ADSCs were induced to undergo chondrogenic differentiation in a chondrogenic differentiation medium using micromass culture with the addition of PBS, CC-EVs, or US-CC-EVs. After 21 days of induction, US-CC-EVs showed enhanced potential to induce chondrogenic differentiation in ADSCs, as evidenced by gross observations, histopathological staining, and the expression of Sox9, COL2, and Aggrecan. We demonstrated that CC-EVs can enhance the chondrogenesis of ADSCs in vitro, and our ultrasound-driven healing enhances the ability of CC-EVs to promote the chondrogenesis of ADSCs.

Based on the finding that US-CC-EVs exhibited better biological effects on ADSCs and promoted chondrogenic differentiation more effectively than CC-EVs, further investigation into the possible underlying mechanisms is warranted. Among the numerous components in EV cargo, miRNAs are crucial constituents that regulate gene expression and modulate cellular function in both healthy and pathological contexts [40,41]. Further exploration of the different miRNAs present in US-CC-EVs and CC-EVs and their potential target genes may provide insights into the underlying mechanisms responsible for their enhanced biological effects. Therefore, we performed miRNA sequencing of US-CC-EVs and CC-EVs and identified differentially expressed miRNAs between the two groups. The sequencing results indicate that US-CC-EVs are enriched with chondrogenesis-related miRNAs, including miR-374a-5p, miR-17-5p, miR-136-5p, miR-455-5p, and miR-140-5p. Zhang et al. revealed the dual functions of miR-17 in maintaining cartilage homeostasis and protecting against osteoarthritis [42]. Chen et al. reported that mesenchymal stem cell-derived exosomal miR-136-5p inhibits chondrocyte degeneration in traumatic osteoarthritis by targeting ELF3 [43]. Tao et al. reported that exosomes derived from miR-140-5p-overexpressing human synovial mesenchymal stem cells enhanced cartilage tissue regeneration and prevented osteoarthritis [44]. GO analysis revealed that the differentially expressed miRNAs were involved in several processes related to cartilage regeneration. KEGG analysis indicated that the improved bioactivity effects of US-CC-EVs could be attributed to different factors associated with focal adhesion, regulation of the actin cytoskeleton, ECM-receptor interaction, PI3K-AKT signaling pathway, cAMP signaling pathway, MAPK signaling pathway, and glycosaminoglycan biosynthesis signaling pathways. These pathways have been reported to contribute to cartilage regeneration [45,46,47,48,49,50]. The enrichment of the aforementioned miRNAs and signaling pathways in US-CC-EVs may elucidate the mechanism behind how low-intensity ultrasound enhances the chondrogenic potential of chondrocyte-derived extracellular vesicles. Additionally, recent reports showed that proteins in EVs may play functional roles [51], so proteomic approaches will also be conducted in the future.

We demonstrated that LIUS for chondrocytes can increase EV secretion by 16-fold and enhance their ability to promote ADSCs chondrogenesis in vitro. Previous studies showed that ADSCs were required to undergo prolonged in vitro chondrogenic induction before transplantation in vivo. In our future studies, we intend to explore if US-CC-EVs can enhance the cartilage repair potential of ADSCs in vivo. We will also attempt to directly apply LIUS in animal models to investigate whether our ultrasound-driven healing can also facilitate cartilage repair by stimulating chondrocyte vesicle secretion. Collectively, this study made the following innovative contributions: (1) we demonstrated that CC-EVs can enhance the chondrogenesis of ADSCs; (2) our study introduces ultrasound-driven healing as an innovative method to enhance both the quantity and quality of chondrocyte-derived vesicles, meeting the clinical demand and addressing the limited chondrogenic potential of ADSCs; and (3) this approach holds the potential to facilitate the direct use of ADSCs for cartilage repair in the future. These findings hold promise for advancing cartilage regeneration strategies and may pave the way for novel therapeutic interventions in regenerative medicine.

## 4. Materials and Methods

### 4.1. Isolation and Culture of Chondrocytes and ADSCs

All animal experiments were conducted in accordance with the National Institutes of Health Guide for the Care and Use of Laboratory Animals. The study was approved by the Animal Research Committee of Shanghai Jiao Tong University Affiliated Ninth People’s Hospital (SH9H-2019-A146-1-1). All the New Zealand white rabbits in this study were purchased from Shanghai Jiao Tong University Agricultural and Biological Experimental Interior Field Co., Ltd. (Shanghai, China). Chondrocytes from the auricular cartilage tissue of rabbits (n = 6) were isolated according to previously established methods [52,53,54,55]. Briefly, the cartilage was excised into 1 mm^2^ pieces. Then the cartilage was digested in high-glucose Dulbecco’s modified Eagle’s medium (DMEM; Gibco, Carlsbad, CA, USA) containing 0.2% collagenase type 2 (Worthington Biochemical, Freehold, NJ, USA) at 37 °C for 8 h. The suspension was filtered through a 100 μm filter, followed by centrifugation at 1500 rpm for 5 min and washed with PBS. The isolated cells were cultured in DMEM supplemented with 10% fetal bovine serum (FBS; Cellmax, Beijing, China) at 37 °C in a humidified atmosphere containing 5% CO_2_. Chondrocytes were passaged using 0.25% trypsin (Gibco, Carlsbad, CA, USA) after 80–90% confluence. The chondrocytes used in the experiments were at passage 2.

Fat obtained from the inguinal region of six rabbits was mixed into a pooled sample for ADSCs isolation as previously described [56]. After isolation, the ADSCs were cultured in low-glucose DMEM (Gibco, Carlsbad, CA, USA) supplemented with 10% FBS (Cellmax, Beijing, China). Cells were cultured at 37 °C in a humidified atmosphere containing 5% CO_2_ and passaged when they reached 80–90% confluence. ADSCs at passage 3 were used for the following experiments.

### 4.2. Characterization of ADSCs

ADSCs at passage 3 were cultured in osteogenic, adipogenic, and chondrogenic differentiation media (Cyagen Biosciences, Guangzhou, China). After incubation, the ADSCs were fixed with 4% paraformaldehyde and subsequently identified using Alizarin Red, Oil Red O, and Alcian Blue staining.

Cell surface marker detection of ADSCs was performed as previously described [57]. ADSCs at passage 3 were diluted at a density of 5 × 10^5^ cells/100 µL in a staining buffer consisting of PBS supplemented with 4% FBS. The cells were then incubated with specific antibodies, including anti-CD31, anti-CD34, anti-CD45, anti-CD34, anti-29, anit-CD90, and anti-CD105 (all direct-labeled antibodies from BD Biosciences, Franklin Lakes, NJ, USA). The incubation was conducted at 4 °C for 30 min. After being washed twice, ADSCs were prepared in 100 µL of staining buffer and analyzed by flow cytometry (CytoFLEX LX, Beckman Coulter, Brea, CA, USA).

### 4.3. Chondrocyte Ultrasound Stimulation

Chondrocytes were stimulated with low-intensity ultrasound using a therapeutic ultrasound apparatus (WEL-100; Well D Medical Electronics Co., Ltd., Shenzhen, Guangdong, China) as previously described [28]. The ultrasound utilized in this study was characterized by a frequency of 1 MHz, a duty cycle of 60%, a pulse repetition frequency of 100 Hz, and an effective area of 2 cm^2^. Specifically, the bottoms of the 6- or 96-well plates or 10-cm culture dishes were coated with a 1 cm layer of ultrasound coupling agent, and the ultrasonic transducer was tightly attached to the bottom of the culture plates or dishes to ensure that there were no air bubbles in between. Different parameters of ultrasonic stimulation were then applied to the chondrocytes (Figure 1A).

### 4.4. Cell Viability Assay

Cell Counting Kit-8 (CCK-8; Dojindo Laboratories, Kumamoto, Japan) was used to assess cell viability in accordance with the kit’s instructions. Chondrocytes (passage 2) were seeded into 96-well plates at a density of 3×10^3^ cells/well and treated with or without ultrasound stimulation. After 3 days, the chondrocytes were incubated in low-glucose DMEM supplemented with 10% CCK-8 solution for 2 h. Then, the absorbance was measured by the microplate reader (Thermo Fisher Scientific Inc., Waltham, MA, USA) at 450 nm. Cell viability was determined using the following formula:

Chondrocyte viability (fold change of control) = (absorbance in the treatment group)/(absorbance in the control group).

### 4.5. Apoptosis Assay

Apoptosis assays were conducted following established protocols. [28]. For the apoptosis assay, chondrocytes (passage 2) were seeded in 6-well plates. After reaching 80% confluence, chondrocytes were washed twice with PBS and treated with high-glucose DMEM in the presence or absence of ultrasound stimulation. After 48 h, apoptosis was assessed using the Annexin V-FITC/PI Apoptosis Detection Kit (#556,547; BD Biosciences, Franklin Lakes, NJ, USA) according to the kit’s instructions. Apoptosis was measured using flow cytometry (CytoFLEX LX; Beckman Coulter, Brea, CA, USA). The flow cytometry settings were optimized for the PE and FITC channels, and the percentage of apoptotic cells was quantified.

### 4.6. Preparation of EVs

After reaching 80% confluence, chondrocytes at passage 2 were rinsed with PBS and cultured in high-glucose DMEM. Chondrocytes were stimulated with or without low-intensity ultrasound as described above. After 48 h, CC-EVs or US-CC-EVs were isolated and purified from the conditioned medium as previously described [18]. The conditioned medium was processed to remove cellular debris through centrifugation at 3000× *g* for 30 min at 4 °C. The remaining cells and debris were eliminated by filtration with 0.45-μm and 0.22-μm filters (Steri-topTM, Millipore, USA). EVs were isolated and concentrated using 100 kDa ultraclear tubes (Millipore Corp., Billerica, MA, USA). The obtained EVs were stored at −80 °C for the following experiments. The characterization of EVs included NTA analysis (ZetaView PMX 110, Particle Metrix, Meerbusch, Germany), transmission electron microscopy (TEM; JEOL microscope, JSM-7001TA, Tokyo, Japan), and western blotting. The yield of EVs was quantified using NTA and BCA analysis by the BCA Protein Assay Kit (#P0010; Beyotime, Shanghai, China).

### 4.7. Cellular Uptake of EVs

The cellular uptake of EVs was assessed as previously described [57]. Briefly, EVs were labeled with CellTracker CM-Dil Dye (#C7000, Invitrogen, Waltham, MA, USA) stock solution (1 mg/mL). The labeled EVs were initially incubated at 37 °C for 5 min and then at 4 °C for 15 min. After incubation, the CM-Dil-labeled EVs were washed with PBS using 100 kDa ultraclear tubes (Millipore Corp., Billerica, MA, USA) three times at 3000 g and 4 °C for 10 min to remove the unbound CM-Dil. ADSCs were incubated with the CM-Dil-labeled EVs (150 μg/mL) for 12 h. Then, the cells were washed three times with PBS, fixed with 4% paraformaldehyde, and stained with phalloidin and 4′,6-diamidino-2-phenylindole (DAPI) (#40732ES10; Yeasen Biotechnology, Shanghai, China). The uptake of labeled EVs by cells was observed with a Nikon A1 confocal microscope (Nikon, Tokyo, Japan).

### 4.8. Western Blotting

After the total EV protein concentration was determined using the BCA protein assay, 30 µg of total protein was loaded on a 10–15% SDS-PAGE gel and subjected to electrophoresis. The separated proteins were transferred to PVDF membranes. Then, the membranes were incubated with primary anti-CD63 (1:1000, #ab134045, Abcam, Cambridge, UK), anti-CD81 (1:1000, #ab109201, Abcam, Cambridge, UK), and anti-TSG101 (1:1000, #ab125011, Abcam, Cambridge, UK). The membranes were subsequently incubated with secondary antibodies (1:5000, #111-035-045; Jackson ImmunoResearch, Madison, WI, USA). Protein expression was visualized using a BeyoECL Plus kit (#MA0186; Meilunbio, Dalian, Liaoning, China).

### 4.9. In Vitro Cell Proliferation Assay

ADSCs at passage 3 were co-cultured with EVs at 50 or 150 g/mL for 2 days. Cell proliferation was assessed using an EdU Cell Proliferation Kit (#C10310-3, Cell-Light EdU Apollo488 In Vitro Kit, Ribo Biotechnology, Guangzhou, Guangdong, China), following the manufacturer’s instructions. Proliferating cells were visualized using a fluorescence microscope (Carl Zeiss, Jena, Germany). Five randomly selected images from each well were analyzed, and the number of EdU-positive cells was quantified using ImageJ software version 2.3.0. The proliferation rate was calculated by the following formula:

Proliferation rate = (Number of EdU − positive cells/total number of cells) × 100%.

### 4.10. Scratch Assay

ADSCs at passage 3 were seeded in a 6-well plate with a density of 4 × 10^5^ cells/well and allowed to reach 100% confluence. Subsequently, a scratch was created across the cell monolayer using a 200-μL pipette tip, and the cells were washed three times with PBS. The control group was cultured in serum-free DMEM; however, the other groups were cultured in serum-free DMEM supplemented with 50 or 150 μg/mL EVs. Images were captured at 0 and 12 h using an inverted microscope (Olympus, Tokyo, Japan), and the area of the uncovered region was measured by the ImageJ software 2.3.0. The cell migration rate was calculated using the following formula:

Migration rate = (scratch area at 0 h − scratch area at 12 h)/(scratch area at 0 h) × 100%

### 4.11. In Vitro Chondrogenic Differentiation of ADSCs

ADSCs were induced to undergo chondrogenic differentiation using micromass culture according to a previously established method [58]. Briefly, ADSCs at passage 3 were resuspended at a concentration of 2 × 10^7^ cells/mL in the chondrogenic medium (#GUXMD_90041; Cyagen Biosciences, Guangzhou, China). Droplets of the cell suspension (12.5 μL) were carefully placed in individual wells of a 24-well plate and incubated at 37 °C for 90 min to allow the cells to adhere to the plate. Next, the droplets were divided into three groups: the CC-EVs group, cultured in 500 μL of chondrogenic medium supplemented with CC-EVs to achieve the EV concentration of 150 μg/mL; the US-CC-EVs group, cultured in 500 μL of chondrogenic medium supplemented with US-CC-EVs to achieve EV concentration of 150 μg/mL; and the PBS group, cultured in 500 μL of chondrogenic medium supplemented with an equal volume of PBS as used in the aforementioned groups, serving as a control. Chondrogenic medium supplemented with EVs or PBS was refreshed every 3 days for each group. After 21 days of inducing chondrogenesis in ADSCs, microspheres from different groups were observed. They were then captured using an inverted microscope (Olympus, Tokyo, Japan), and the size of the microspheres was measured using ImageJ software 2.3.0. Samples were collected for further experiments.

### 4.12. Histological Analyses and Immunofluorescence Staining

After 21 days of chondrogenesis, the microspheres were rinsed with PBS and fixed in 4% paraformaldehyde for 24 h. The samples underwent dehydration using a series of alcohol solutions, followed by embedding in paraffin and perpendicular sectioning into 5 μm-thick slices. Histological detection was conducted using H&E, safranin O, and Alcian blue staining methods.

For immunofluorescence staining, sections were incubated with primary anti-Col2 (1:200, #ab185430, Abcam) or anti-Sox9 (1:200, #ab185966, Abcam) antibodies. The sections were then incubated with FITC-conjugated goat anti-mouse IgG (1:500, #115-095-003; Jackson ImmunoResearch, Madison, WI, USA) or FITC-conjugated goat anti-rabbit IgG (1:500, #111-095-003; Jackson ImmunoResearch). The cell nuclei were stained with DAPI (1:1000, #AR1176, Boster, Wuhan, China). Finally, the stained sections were examined under a fluorescence microscope (Carl Zeiss, Jena, Germany).

### 4.13. Quantitative RT-PCR Analysis

After a 21-day chondrogenesis period, the total RNA was isolated using a Tissue RNA Purification Kit Plus (EZBioscience, Roseville, MN, USA) following the manufacturer’s instructions. cDNA synthesis was carried out using a Reverse Transcription Master Mix (EZBioscience). For qRT-PCR, the CFX384 Touch Real-Time PCR Detection System (Bio-Rad, Hercules, CA, USA) was employed along with SYBR Green qPCR Master Mix (ROX2 plus, EZBioscience). The qPCR procedure consisted of an initial hot start at 95 °C for 5 min, followed by 40 cycles of denaturation at 95 °C for 10 s, and annealing/extension at 60 °C for 30 s. The expression levels of glyceraldehyde 3-phosphate dehydrogenase (GAPDH) were used as internal controls to normalize gene expression. The relative mRNA expression levels were determined using the 2^−ΔΔCT^ method, and the results were presented as fold-increases compared to the control samples. The primer sequences are listed in Table 1.

### 4.14. RNA Extraction and Library Construction

Total RNA was extracted by the TRIzol reagent (Invitrogen, Waltham, MA, USA) according to the manufacturer’s instructions. The concentration of total RNA was determined using the Nanodrop 2000 (Thermo Fisher Scientific Inc., Waltham, MA, USA), and the integrity of the RNA samples was assessed using the Agilent 2100 Bioanalyzer (Agilent Technology, Santa Clara, CA, USA).

For small RNA library construction, 1 μg of total RNA from each sample was used with the NEBNext Small RNA Library Prep Set for Illumina kit (Cat. No. NEB#E7330S, NEB, San Diego, CA, USA) according to the manufacturer’s recommendations. In brief, the total RNA was ligated with adapters at both ends, followed by reverse transcription to cDNA and PCR amplification. The PCR products ranging from 140 to 160 bp were isolated and purified to obtain small RNA libraries. The quality of the libraries was assessed using the Agilent Bioanalyzer 2100 system. Finally, the libraries were sequenced on the Illumina Novaseq 6000 platform, generating 150 bp paired-end reads. The small RNA sequencing and analysis were performed by OE Biotech Co., Ltd. (Shanghai, China).

### 4.15. Bioinformatics Analysis

The raw data/reads obtained from base calling were processed to obtain high-quality clean reads. This involved filtering out low-quality reads, removing reads with 5′ primer contaminants and poly(A) sequences, and eliminating reads without 3′ adapters and insert tags. The resulting clean reads were then analyzed.

The length distribution of the clean sequences in the reference genome was determined. The sequences were aligned using Bowtie software [59], and various RNA types such as rRNA, scRNA, Cis-reg, snRNA, tRNA, and others were annotated and filtered using the Rfam v.10.1 database (http://www.sanger.ac.uk/software/Rfam, accessed on 9 September 2023) [60]. The cDNA sequences and species repeat sequences from the Repbase database were identified using Bowtie software. The mature miRNAs were identified by aligning the clean reads against the miRBase v22 database [61]. The expression patterns of miRNAs in different samples were analyzed. Additionally, the unannotated reads were analyzed using miRDeep2 [62] to predict novel miRNAs. Based on the hairpin structure of a pre-miRNA and the information in the miRBase database, the corresponding miRNA star sequence and miRNA mature sequence were identified.

Differentially expressed miRNAs were identified according to the criteria of q value < 0.05 and fold change (FC) > 2 or FC < 0.5. The q value was calculated by the DEG algorithm in the R package. The target genes of differentially expressed miRNAs were predicted using the miRanda software [63] for animal miRNAs. The parameters used were S ≥ 150, ΔG ≤ -30 kcal/mol, and a strict 5′ seed pairing requirement.

GO enrichment and KEGG pathway enrichment analyses for differentially expressed miRNA-target genes were performed using R based on the hypergeometric distribution.

### 4.16. Statistical Analysis

The numerical data are expressed as mean ± standard deviation and analyzed using one-way analysis of variance (ANOVA) followed by Tukey’s post hoc test. Statistical analysis was conducted using GraphPad Prism version 9.5.0 (GraphPad Software, San Diego, CA, USA). Statistical *p*-values less than 0.05 (*p* < 0.05) were considered statistically significant, indicating a significant difference among the groups.

## Figures and Tables

**Table 1:** Primers for real-time RT-PCR.

Target Genes	Forward	Reverse
GAPDH	ATGGTGAAGGTCGGAGTGA	AACATCCACTTTGCCAGATTA
Aggrecan	CACCCCGGAATCAAATGGA	TGGGCAGCGAGACCTTGT
Col2	TCCTGTGCGAGACATAATCT	GCAGTGGCGAGGTCAGTAG
Sox9	GGCTCCGACACCGAGAATA	TCCTCTTCGCTCTCCTTCTTG

## Data Availability

The datasets generated and analyzed in the current study are available from the corresponding author upon reasonable request.

## Supplementary Materials

The following supporting information can be downloaded at: https://www.mdpi.com/article/10.3390/biomedicines11102836/s1, Figure S1: Characterization of ADSCs.
