# Supplementary material for: Ultrasound-Driven Healing: Unleashing the Potential of Chondrocyte-Derived Extracellular Vesicles for Chondrogenesis in Adipose-Derived Stem Cells
Source: Biomedicines. 2023 Oct 19;11(10):2836. doi: 10.3390/biomedicines11102836 (PMC10604747; doi:10.3390/biomedicines11102836)
Supplement: Supplementary file 1 [file biomedicines-11-02836-s001.zip › biomedicines-2631174-SI.pdf]

Supplementary Materials

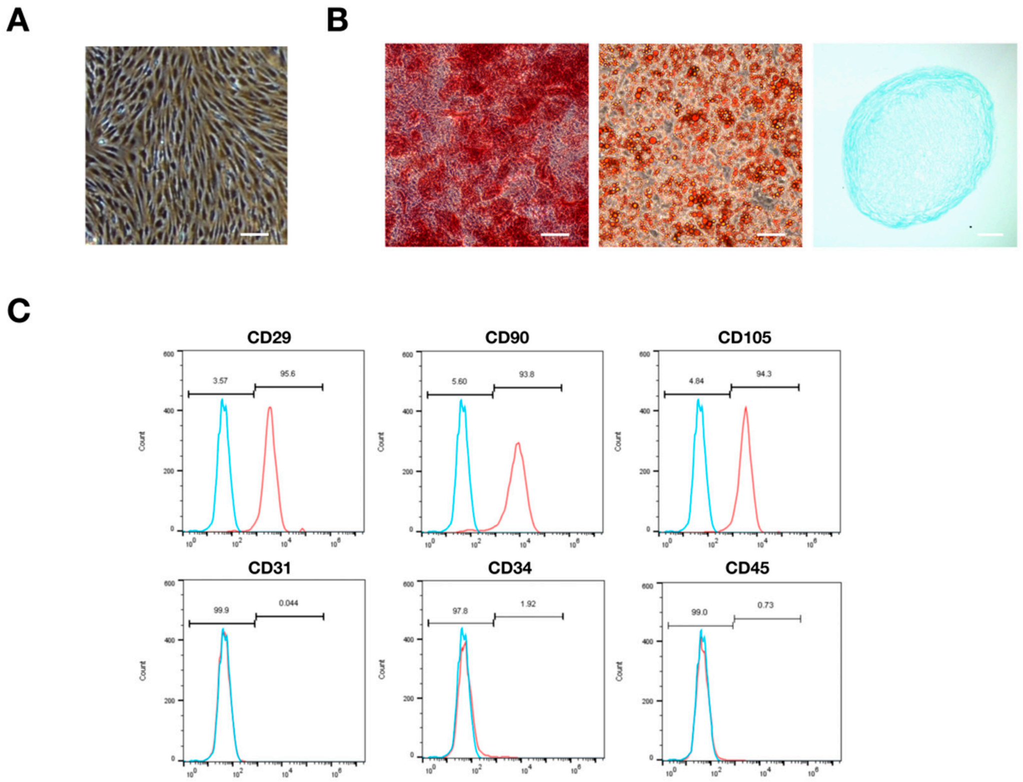


**Figure S1.** Characterization of ADSCs. (A) The morphology of ADSCs by light microscopy; scale bar = 100 μm. (B) ADSC osteogenic, adipogenic, and chondrogenic differentiation; scale bar = 200 μm. (C) Surface marker expression of ADSCs evaluated by flow cytometry.
